# Morbidity and cost burden of methicillin-resistant *Staphylococcus aureus *in early onset ventilator-associated pneumonia

**DOI:** 10.1186/cc4934

**Published:** 2006-06-29

**Authors:** Andrew F Shorr, Ying P Tabak, Vikas Gupta, RS Johannes, Larry Z Liu, Marin H Kollef

**Affiliations:** 1 Pulmonary and Critical Care Medicine Service, Washington Hospital Center, Washington, District of Columbia, USA; 2 Cardinal Health Clinical – Research Group, 500 Nickerson Road, Marlborough, Massachusetts, USA; 3 Pfizer Inc, New York, New York, USA; 4 Washington University School of Medicine, St. Louis, Missouri, USA

## Abstract

**Introduction:**

To gain a better understanding of the clinical and economic outcomes associated with methicillin-resistant *Staphylococcus aureus *(MRSA) infection in patients with early onset ventilator-associated pneumonia (VAP), we retrospectively analyzed a multihospital US database to identify patients with VAP over a 24 month period (2002–2003).

**Method:**

Data recorded included physiologic, laboratory, culture, and other clinical variables from 59 institutions. VAP was defined as new positive respiratory culture after at least 24 hours of mechanical ventilation (MV) and the presence of primary or secondary ICD-9-CM diagnosis codes of pneumonia. Outcomes measures included in-hospital morbidity and mortality for the population overall and after onset of VAP (duration of MV, intensive care unit [ICU] stay, in-hospital stay, and case mix and severity-adjusted operating cost). The overall cost was calculated at the hospital level using the Center for Medicare and Medicaid Services Cost/Charge Index for each calendar year.

**Results:**

A total of 499 patients were identified as having VAP. *S. aureus *was the leading organism (31% of isolates). Patients with MRSA were significantly older than patients with methicillin-sensitive *Staphylococcus aureus *(MSSA; median age 74 versus 67 years, *P *< 0.05) and more likely to be medical patients. Compared with MSSA patients, MRSA patients on average consumed excess resources of 4.4 (95% confidence interval 0.6–8.2) overall MV days, 3.8 (-0.5 to +8.0) days of inpatient length of stay (LOS), 5.3 (1.0–9.7) ICU days, and US$7731 (-US$8393 to +US$23,856) total cost after controlling for case mix and other factors. Furthermore, MRSA patients needed excess resources after the onset of VAP (4.5 [95% confidence interval 1.0–8.1] MV days, 3.7 [-0.5 to +8.0] inpatient days, and 4.4 [0.4–8.4] ICU days) after controlling for the same case mix and admission severity covariates.

**Conclusion:**

*S. aureus *remains a common cause of VAP. VAP due to MRSA was associated with increased overall LOS, ICU LOS, and attributable ICU LOS compared with MSSA-related VAP. Although not statistically significant because of small sample size and large variation, the attributable excess costs of MRSA amounted to approximately US$8000 per case after controlling for case mix and severity.

## Introduction

Ventilator-associated pneumonia (VAP) is a major nosocomial infection in the intensive care unit (ICU), affecting between 10% and 20% of patients who receive more than 48 hours of mechanical ventilation (MV) [1]. Several studies [2-5] have documented that VAP significantly increases hospital and ICU length of stay (LOS), duration of MV, and hospital costs. VAP may also increase mortality, although this remains controversial [6].

*Staphylococcus aureus *is the leading cause of VAP in Europe and ranks alongside *Pseudomonas aeruginosa *as the most prevalent cause of VAP in US hospitals [6,7]. The increasing prevalence of antibiotic-resistant strains is of concern, with more than 60% of *S. aureus *isolates recovered from the ICU now resistant to methicillin (methicillin-resistant *Staphylococcus aureus *[MRSA]) [8]. MRSA appears to be associated with worse outcomes than methicillin-sensitive *S. aureus *(MSSA) infection. In a meta-analysis conducted by Cosgrove and coworkers [9], MRSA bacteremia was found to increase significantly the risk for death compared with MSSA bacteremia. However, evaluation of the excess burden of MRSA compared with MSSA has also been limited by confounding factors. In one of the largest studies evaluating the impact of MRSA in VAP [10], the unadjusted mortality rate was significantly higher for MRSA than for MSSA infection. Following adjustment for severity of illness at admission, duration of ICU LOS before VAP diagnosis, and adequacy of antimicrobial treatment, this difference was no longer significant. Combes and coworkers [11] recently evaluated a group of VAP patients who all received initial appropriate antibiotic therapy, and reported that there was no excess attributable mortality for MRSA versus MSSA.

Although mortality is an important end-point in any analysis of the critically ill, morbidity and costs of critical care are also significant issues [12]. Investigators in the US have reported that MRSA significantly increases LOS and hospital charges compared with MSSA in bacteremia and surgical site infections, with an excess attributable LOS of 2–8 days and excess attributable charges of US$7000–14,000 [13-15]. Morbidity and cost data from analyses of multiple institutional databases specific to MRSA-related pneumonias are more limited and are not US focused.

Given the paucity of local data on the clinical and economic impact of MRSA-related VAP, we conducted a retrospective analysis involving a large US database with two objectives in mind: to identify risk factors associated with the development of VAP among patients admitted to the ICU and to assess the affect of VAP on patient outcomes, including overall and attributable hospital morbidity, mortality, and total costs.

## Materials and methods

### Study design

A retrospective cohort analysis was performed to examine the clinical and economic outcomes associated with MRSA and MSSA in patients with VAP. All available clinical and financial data were obtained for all patients with VAP admitted to 59 US hospitals (16 teaching and 43 nonteaching) between 1 January 2002 and 31 December 2003.

Analyses of the impact of pathogens on MRSA versus MSSA mono-microbial subgroups (total *n *= 154) were particularly focused on the objective measures of resource utilization, including length of MV, in-patient LOS, and ICU LOS. These dependent variables were analyzed separately as the overall length of event and the length of event from the onset of VAP. Hospital costs for each VAP case were calculated as follows: specific operating cost (defined as the hospital specific cost/charge ratio × hospital total charges); and total cost (defined as [operating cost/charge ratio + capital cost/charge ratio] × total charge). The cost/charge ratios were obtained from the Center for Medicare and Medicaid Services database for each hospital for calendar years 2002 and 2003 [16]. In other words, we computed total costs for each patient by taking the component charges for the hospital stay (for example, pharmacy, laboratory, bed day, and so on) and then multiplying them by the Medicare cost-to-charge ratios. These component costs were then summed to derive the total costs.

### Data source

Data for the present analysis were obtained from the Cardinal Health Research Database (Cardinal Health, MediQual, Marlborough, MA, USA [formerly MedisGroup]) [3], a large, multi-institutional database of US acute-care hospitals. Details of this database were reported previously [3,17-20]. Briefly, the database comprises clinical findings, including patient's clinical history, pathophysiologic findings (such as, vital signs, laboratory test results, culture findings), and physician assessments also includes administrative data imported from hospital information systems [3,7,17-20]. The clinical data are generally collected for the first two days of hospitalization for all participating hospitals on all of the patients. For a small group of hospitals (*n *= 59), laboratory data, including culture data, are collected for the first five days of hospitalization. Hospitals that opted for five day data collections did so voluntarily, primarily for the purposes of internal quality improvement. Because of this limitation, we were only able to identify early onset VAP (mostly within five days of hospitalization).

### Study sample

During the study period, a total of 59 US hospitals (16 teaching and 43 nonteaching) collected clinical and culture data for the first 5 days for all of their patients. Among the 59 hospitals, 42 reported early onset VAP cases with positive culture (within five days of hospitalization). These 42 hospitals were included in the final analysis. Among them, 17% had numbers of beds below 100; 76% had between 101 and 300 beds, and 7% had more than 300. The teaching hospitals accounted for 21% of hospitals. VAP was defined as present in patients who were on a ventilator for at least 24 hours with a first positive bacterial respiratory culture after initiation of MV and with either primary or secondary ICD-9-CM (International Classification of Diseases, Ninth Revision, Clinical Modification) diagnosis codes for pneumonia. For patients who had more than one admission to the ICU for VAP during the same hospitalization, the first ICU admission was used in this analysis. Resource utilization (in-hospital LOS, ICU LOS, and duration of MV) included data for the entire hospital stay.

### Statistical analysis

Univariate and multivariate analyses were conducted for all dependent measures. Nonparametric tests were used for continuous variables and χ^2 ^test for dichotomous variables. Ordinary least square multiple regressions were used to estimate the impact of MRSA on resource utilizations. Covariates included risk for death score (the predicted probability of death based on admission severity), severe trauma, major surgery, number of days from admission to onset of VAP, and MRSA. The risk factors used to generate the death propensity score included demographic variables, coexisting conditions (such as, cancer, cerebrovascular disease, liver disease, renal disease, congestive heart failure, immunosuppressed status), physical examination findings (for example, vital signs, altered mental status), laboratory findings (for example, blood urea nitrogen, glucose), and other clinical findings. In the multivariate analysis we also tested the effect of mortality status to examine whether patients who died in hospital had different resource consumption compared with survivors. In addition, we tested the interaction effect of mortality status and MRSA status to determine whether the MRSA effect varied by mortality status. Finally, we tested hospital's teaching status on all of the outcome measures.

All statistical analyses were conducted using SAS software (version 9.01; SAS Institute Inc., Cary, NC, USA). *P *< 0.05 was considered statistically significant.

## Results

### Patient characteristics, pathogen distribution, and risk factors for ventilator-associated pneumonia

**Figure 1:**
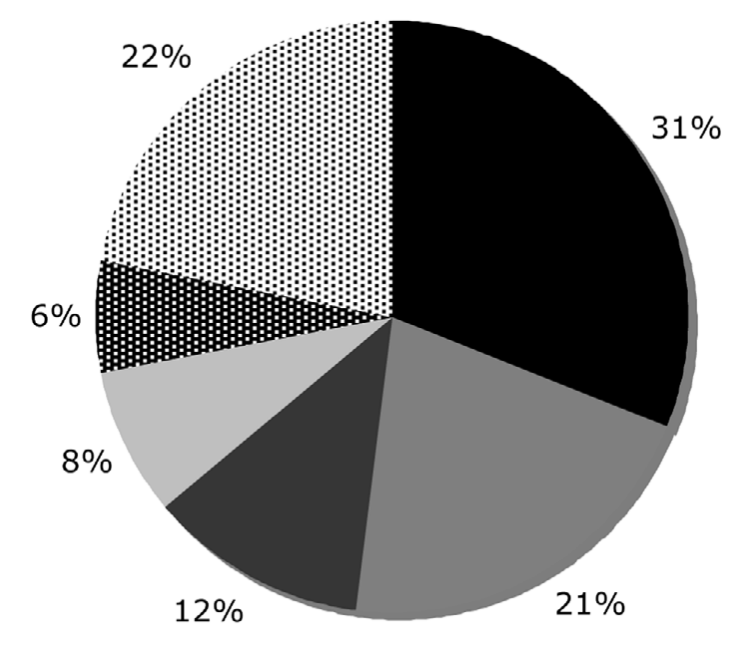
Etiologic agents of ventilator-associated pneumonia: frequencies. *Staphylococcus aureus *(31%) was the most frequent organism isolated in patients with a confirmed diagnosis of ventilator-associated pneumonia followed by *Pseudomonas aeruginosa *(21%), *Haemophilus influenzae *(12%), *Klebsiella pneumoniae *(8%), and *Escherichia coli *(6%). Other identified organisms accounted for 22% of all isolates.

A total of 499 patients were identified as having VAP between 1 January 2002 and 31 December 2003. Of mono-microbial respiratory cultures, *S. aureus *was the most frequent organism isolated in patients with a confirmed diagnosis of VAP (31%; Figure 1), followed by *P aeruginosa *(21%), *Haemophilus influenzae *(12%), *Klebsiella pneumoniae *(8%), and *Escherichia coli *(6%). Other identified organisms accounted for 22% of the total isolates. A total of 95 patients had MSSA-related VAP, 59 had MRSA-related VAP, and 182 patients were infected with a Gram-negative organism.

There were significant differences in demographics and clinical characteristics among patients with VAP due to MRSA, MSSA, and Gram-negative organisms (Table 1). Compared with patients with VAP due to MSSA or Gram-negative organisms, patients with VAP due to MRSA were older, were more likely to have congestive heart failure or immunosuppression as a comorbidity, were more likely to be medical patients, and were less likely to be admitted to a trauma or surgical unit. They more likely to be covered by Medicare. Compared with patients with VAP due to a Gram-negative organism, patients with MRSA-related VAP were more likely to be admitted with a pleural effusion and more likely to have diabetes mellitus. However, acute clinical presentations represented by laboratory findings, vital signs, Glasgow Coma Scale score, and presence of bacteremia, as well as aggregated severity of illness measured by Admission Severity Group, were generally similar across the three groups (Table 1).

### Clinical and economic outcomes

In patients with VAP, the crude in-hospital mortality rate of patients with *S. aureus *infection was not statistically significantly different from that due to Gram-negative infection (29% versus 36%). Similarly, there were no significant differences in terms of mortality rates between patients with MRSA and those with MSSA (29% versus 36%).

**Figure 2:**
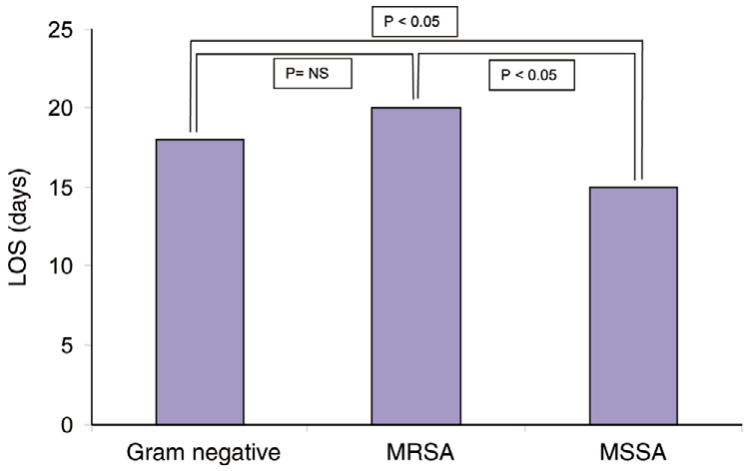
Length of stay for MRSA, MSSA, and Gram-negative infections. Patients with MRSA (20 days) or Gram-negative (18 days) related VAP had a longer overall median LOS than did patients with MSSA-related (15 days) VAP. MRSA, methicillin-resistant *Staphylococcus aureus*; MSSA, methicillin-sensitive *Staphylococcus aureus*; LOS, length of stay; VAP, ventilator-associated pneumonia.

Patients with MRSA-related VAP had a longer overall LOS (20 versus 15 days; *P *= 0.04) and ICU LOS (13 versus 9 days; *P *< 0.006) than did patients with MSSA-related VAP (Table 2, Figure 2). The univariate analysis stratified by mortality status indicated that MRSA survivors consumed more resources than did MSSA survivors. In contrast, MSSA patients who died consumed more resources than did MRSA patients who died. This reversal effect of MRSA by mortality status may be partly due to the case mix; more MSSA patients were trauma or surgical patients whereas more MRSA patients were older and sicker medical patients who died faster, thus consuming less resources.

The multivariate analysis indicated that MRSA patients consumed excess resources of 4.4 overall days on MV (*P *= 0.03), 3.8 inpatient days (*P *= 0.08), 5.3 days in the ICU (*P *= 0.02), and US$7731 in total costs (*P *= 0.35) after controlling for surgical and trauma status, time of onset of VAP, and admission severity compared with MSSA patients (Table 3). Furthermore, the MRSA patients consumed similar amount of excess resources after the onset of VAP, including VAP MV days, VAP inpatient LOS, and VAP ICU days compared with MSSA (Table 3). When we added mortality status and the interaction of mortality and MRSA status to the model, we find them significant only for the total ICU length of stay model. Also, the results were similar for all models. Hospital teaching status was strongly correlated with major surgery and trauma status, and was therefore eliminated from the final model.

## Discussion

The present investigation utilized a large US database to analyze retrospectively the clinical and health economic impact of VAP caused by MRSA versus MSSA infection. We found that MRSA-related VAP was associated with significantly increased overall hospital LOS, ICU LOS, and attributable ICU LOS as compared with MSSA. Although not statistically significant because of small sample size and large variance, the attributable excess cost reached approximately US$8000 per case after controlling for case mix and severity.

The present analysis of objectively measured resource utilization and hospital level costs for the overall and attributable impact of MRSA confirms the findings of two other retrospective inpatient US database analyses [7,21]. Kollef and coworkers [7] observed that *S. aureus *was a major pathogen not only in VAP but also in community-acquired, hospital-acquired, and health care associated pneumonias. In that previous study, pneumonias acquired in the health care and hospital settings were associated with greater LOS and increased cost compared with community-acquired pneumonia, with *S. aureus *found to be the only pathogen that correlated with mortality after controlling for confounding factors [7]. Noskin and colleagues [21] reported that *S. aureus *infection increased the average hospital LOS by threefold, with five times the risk for in-hospital mortality and threefold greater hospital charges compared with inpatients without *S. aureus *infection. The latter study exclusively relied on ICD-9-CM codes, which did not differentiate between MRSA and MSSA, whereas our study used pathophysiologic data (culture) to confirm the pathogens. Furthermore, the study conducted by Noskin and coworkers used ICD-9-CM codes for severity adjustment, whereas our study used clinical data (vital signs, laboratory, and other clinical findings) for more accurate severity adjustment. Finally, the clinical database we used allowed us to assess the morbidity impact of MRSA after the onset of VAP.

Compared with a recent study using clinical data investigating the impact of MRSA on VAP in France [22], which showed that MRSA prolonged the median ICU LOS by 11 days compared with MSSA, independent of illness severity, the present study in the US patient population found shorter excess ICU LOS (about four days) after controlling for confounders. This might be due to possible differences in VAP case mix between the two studies and in ICU management between the USA and France.

Measuring the impact of MRSA is crucial for clinicians and administrators aiming to understand the added resource use arising from its diagnosis, prevention, and treatment. Conceivably, even minimally effective preventive strategies could lead to substantial cost savings, and approaches that combine infection prevention and decreasing the prevalence of MRSA have the potential to yield major savings. In the case of MRSA-related VAP, silver coated endotracheal tubes that purportedly prevent VAP or lymphostatin, which is used to prevent patient colonization by MRSA, may find roles in the critical care setting pending successful demonstration of efficacy in clinical trials [23,24]. Although measures to control the spread of MRSA may seem cumbersome to providers, the findings of this and other studies suggest that any costs associated with the implementation of such measures may quickly be offset by cost savings in the ICU [25,26]. Any therapeutic intervention directed against nosocomial MRSA infection should be one component of a comprehensive strategy that includes hygiene controls, early pathogen detection, isolation of MRSA-infected patients, and elimination of inappropriate antibiotic use [26]. With respect to the latter, drug acquisition costs are a small component of overall hospital costs and may in fact be trivial compared with the costs associated with prolonged ICU LOS as a result of initial inadequate empiric antibiotic selection.

Our analysis has several limitations. First, we only identified early onset VAP cases within five days of hospitalization. This probably underestimated the prevalence of VAP and the impact of MRSA because many VAP cases are likely to develop later during hospitalization. Second, the retrospective design is associated with a number of limitations, and the present study is as subject to these as any study of this design. An example is that coding practices relating to the use of the ICD-9-CM system vary across hospitals and may be influenced by financial incentives. This introduces uncertainty and limits our findings, despite the fact that we used the concomitant presence of positive respiratory culture and ICD-9-CM codes to define the VAP cases. Third, because we used positive culture to define cases, we were unable to estimate the potential VAP cases in general and MRSA cases in particular. Fourth, given the unique aspects of the hospitals we studied and the other issues noted above, the generalizability of our findings may be limited. Finally, we could not assess the possible confounding factor of appropriate use of antibiotics because this information was not available.

## Conclusion

Despite the limitations outlined above, the present study highlights the clinical and economic importance of MRSA in VAP. The data suggest that a diagnosis of MRSA-related VAP is an important determinant of excess hospital and ICU LOS, and attributable costs compared with MSSA infection. These data support the need to develop and implement effective strategies for the diagnosis, treatment, and prevention of MRSA-related VAP in order to reduce patient morbidity and costs associated with provision of health care.

## Key messages

• 	Morbidity and cost data from analyses of large, multi-institutional databases specific to MRSA infection in the USA are limited.

• 	We quantified the clinical and economic outcomes associated with MRSA infection in patients with early onset VAP.

• 	*S. aureus *was found to be a common cause of VAP. Compared with VAP due to MSSA, VAP due to MRSA was associated with increased overall and attributable resource utilization, prolonged length of stay, and total costs of hospitalization.

## Abbreviations

ICD-9-CM = International Classification of Diseases, Ninth Revision, Clinical Modification; ICU = intensive care unit; LOS = length of stay; MRSA = methicillin-resistant *Staphylococcus aureus*; MSSA = methicillin-sensitive *Staphylococcus aureus*; MV = mechanical ventilation; VAP = ventilator-associated pneumonia.

## Competing interests

AFS and MHK serve as consultants and have received speaking fees from Pfizer. LZL is an employee of Pfizer. All other authors have no competing interests to disclose.

## Authors' contributions

AFS, YPT, VG, RSJ, LZL, and MHK were involved in development of the study concept and design and analysis and interpretation of data. YPT, AFS, and LZL provided statistical expertise. AFS, YPT, and VG drafted the manuscript and all authors were involved with critical revisions of the manuscript. All authors read and approved the final manuscript.

## Figures and Tables

**Table 1:** Patient demographic and clinical characteristics

Characteristics	MRSA (*n *= 59)	MSSA (*n *= 95)	Gram negative (*n *= 182)
Demographics			
Age (median [IQR]; years)	74 (64–81)	67* (47–78)	65^† ^(53–76)
Male	55.9%	52.6%	63.2%
Insurance			
Medicare	67.8%	49.5%*	50.0%^†^
Medicaid	3.4%	8.4%	6.0%
Managed care	15.3%	17.9%	17.0%
Comorbidities			
Immunosuppressed	22.0%	8.4%*	12.1%^†^
Cancer	15.3%	9.5%	16.5%
Diabetes mellitus	39.0%	28.4%	25.8%^†^
Chronic renal failure	17.0%	9.5%	13.7%
Congestive heart failure	45.8%	22.1%*	19.8%^†^
Median number of comorbidities (IQR)	3 (1–5)	2* (0–4)	2 (1–3)
Acute clinical presentation on admission			
Albumin <2.4 g/dl	3.4%	4.2%	4.4%
Arterial pH <7.3	5.1%	13.7%	8.2%
Creatinine >1.5 mg/dl	8.5%	15.8%	6.6%
WBC <4.3 or >19.8 × 10^3^/mm^3^	6.8%	12.6%	6.6%
Bands >32%	1.7%	2.1%	1.1%
BUN >55 mg/dl	10.2%	5.3%	4.4%
BUN 40–55 mg/dl	5.1%	2.1%	2.2%
Temperature <35.6°C	11.9%	27.4%*	14.8%
Respiration >39 breaths/min	5.1%	4.2%	0.6%^†^
GCS^c ^<6	27.1%	16.8%	20.3%
Pleural effusion	28.8%	20.0%	17.0%^†^
Bacteremia	10.2%	14.7%	7.1%
ASG^d ^≥ 3	72.6%	72.9%	66.5%
Care unit			
Trauma unit	2.4%	13.9%*	15.1%^†^
Major surgery	11.9%	17.9%	26.9%

*P < 0.05 for MSSA versus MRSA; ^†^*P *< 0.05 for Gram-negative versus MRSA. ASG, Admission Severity Group; BUN, blood urea nitrogen; GCS, Glasgow Coma Scale; IQR, interquartile range; MRSA = methicillin-resistant *Staphylococcus aureus*; MSSA = methicillin-sensitive *Staphylococcus aureus*.

**Table 2:** Nonparametric univariate analysis of MRSA versus MSSA: duration of MV, inpatient LOS, ICU LOS, and total cost

Dependent variables	MRSA – all (*n *= 59)	MSSA – all (*n *= 95)	*P *value	MRSA Survivors (*n *= 42)	MSSA Survivors (*n *= 61)	*P *value	MRSA nonsurvivors (*n *= 17)	MSSA nonsurvivors (*n *= 34)	*P *value
Total MV (days)	8 (4–20)	8 (3–14)	0.02	12 (3–21)	7 (3–10)	0.006	5 (4–10)	12 (6–15)	0.28
VAP MV (days)	6 (1–16)	6 (1–10)	0.04	9 (1–16)	3 (1–9)	0.03	3 (3–9)	7 (4–12)	0.38
Total inpatient LOS (days)	20 (11–32)	15 (11–23)	0.04	21 (13–32)	15 (10–25)	0.06	12 (7–29)	16 (13–20)	0.27
VAP inpatient LOS (days)	12 (7–23)	13 (8–18)	0.21	16 (9–26)	13 (8–19)	0.16	5 (3–12)	13 (8–17)	0.46
Total ICU (days)^a^	13 (6–27)	9 (6–16)	0.006	15 (8–29)	7 (4–12)	0.0002	6 (5–12)	13 (9–19)	0.26
VAP ICU (days)^a^	7 (3–16)	7 (3–12)	0.04	11 (7–20)	6 (3–11)	0.006	4 (3–10)	10 (6–15)	0.14
Total cost (US$)	40,734 (18,347–71,064)	36,523 (15,539–72,080)	0.30	41,968 (24,173–74,249)	34,939 (14,959–75,787)	0.30	31,597 (17,820–62,967)	45,854 (18,958–55,578)	0.49

A total of 154 patients were included in this analysis. Values are expressed as median (interquartile range). ^a^Analysis for ICU days was conducted on patients (*n *= 95) from hospitals that collect ICU stay data. ICU, intensive care unit; LOS, length of stay; MRSA, methicillin-resistant *Staphylococcus aureus*; MSSA, methicillin-sensitive *Staphylococcus aureus*; MV, mechanical ventilation; VAP, ventilator-associated pneumonia.

**Table 3:** Multivariate analysis of MRSA versus MSSA: duration of MV, inpatient LOS, ICU LOS, and total cost

Dependent variables	MRSA estimate (95% CI)	*P *value	Model r^2^
Total MV (days)	4.4 (0.6 to 8.2)	0.03	0.16
VAP MV (days)	4.5 (1.0 to 8.1)	0.01	0.10
Total inpatient LOS (days)	3.8 (-0.5 to +8.0)	0.08	0.26
VAP inpatient LOS (days)	3.7 (-0.5 to +8.0)	0.08	0.06
Total ICU (days)^a^	5.3 (1.0 to 9.7)	0.02	0.13
VAP ICU (days)^a^	4.4 (0.4 to 8.4)	0.03	0.10
Total cost (US$)	7731 (-8393 to +23,856)	0.35	0.28

A total of 154 patients were included in this analysis. ^a^Analysis for ICU days was conducted on patients (*n *= 95) from hospitals that collect ICU stay data. CI, confidence interval; ICU, intensive care unit; LOS, length of stay; MRSA, methicillin-resistant *Staphylococcus aureus*; MSSA, methicillin-sensitive *Staphylococcus aureus*; MV, mechanical ventilation; VAP, ventilator-associated pneumonia.
